# Muscle Contraction Adaptations in Top-Level Karate Athletes Assessed by Tensiomyography

**DOI:** 10.3390/ijerph191610309

**Published:** 2022-08-19

**Authors:** Velimir Jeknić, Milivoj Dopsaj, Lazar Toskić, Nenad Koropanovski

**Affiliations:** 1 Palms Sports, Abu Dhabi P.O. Box 111188, United Arab Emirates; 2 Department of Sport, Abu Dhabi Police College, Abu Dhabi P.O. Box 111188, United Arab Emirates; 3 Faculty of Sport and Physical Education, University of Belgrade, 11030 Belgrade, Serbia; 4 Faculty of Sport and Physical Education, University in Priština-Kosovska Mitrovica, 38218 Leposavić, Serbia; 5 Faculty of Sport, University “Union-Nikola Tesla”, 11000 Belgrade, Serbia; 6 Department of Criminalistics, University of Criminal Investigation and Police Studies, 11080 Belgrade, Serbia

**Keywords:** TMG, contraction time, neuromuscular changes, thigh muscles, combat sports, sport-specific adaptations

## Abstract

Background: This paper aimed to compare the involuntary stimulated neuromuscular response of thigh muscles in top-level karate athletes and recreational groups. Methods: The study included 13 male karate athletes (KAs) and 14 non-athlete male individuals (NAs). Tensiomyographic (TMG) measurements were obtained from the rectus femoris (RF), vastus medialis (VM), vastus lateralis (VL), biceps femoris (BF) and semitendinosus (ST). Results: Statistically significant differences were observed between KAs and NAs in knee extensor/flexor delay time (Td), contraction time (Tc), total contraction time (Tct), maximal radial displacement of the muscle belly (Dm) and rate of muscle tension development (RMTD). On a group level, KA dominant-leg extensors and flexors and also non-dominant-leg knee flexors had significant differences when compared to NA. Tct is a TMG parameter in which the KAs and NAs differ the most in the case of the knee extensors, while flexor muscles differ the most in the RMTD parameter. Conclusions: The lower Tct values indicate an improved ability of top-level karateka to make fast contractions of the agonist muscles. KAs’ higher RMTD values suggest on strength characteristics needed in breaking actions of the antagonist muscles. Existence of contraction-relaxation-contraction neuromuscular pattern in the RF muscle suggests on implementation of training strategies that involves both rapid muscle contractions and relaxations.

## 1. Introduction

Karate is considered to be one of the most popular martial arts in the world [1,2,3]. It is classified among specialties requiring high technical skills, such as a fine control of movement, accompanied by a great ability to perform the main technical actions as fast as possible—“ballistic actions” [4]. A karate athlete is continuously challenged in the goal of performing very complex actions, combining high movement velocities with high precision [5]. A karate fight (Kumite) is a real match/combat between two competitors under strict rules; they are free to move, kick and punch in defensive and offensive manners [6]. Karate is a good example of a competitive sport with high levels of temporal and spatial constraints, which require rapid reactions [7].

There has been long-standing interest in combat athletes’ lower-body muscle performance [8,9,10,11,12]. Karate punches, kicks, blocks and frequent movements require a joint action sequence with the participation of the leg, pelvis, torso and arm muscles. Hariri et al. [13] highlighted the importance of leg muscle properties in two cases in karate sport. One is regarding kicks, where they pointed out that increasing the knee speed and minimizing the time elapsed from the ground is effective in improving kicking techniques. The second claim considered movement efficiency, where they emphasized that the ability to execute rapid maneuvers and changes in direction while moving on the ground is of utmost importance in karate performance. When it comes to punching execution, the lower part of the body is also the primary contributor because of the fact that ground force reaction is generated by the legs and transferred to the upper body, allowing for a powerful movement [14,15]. To give an illustration, research by Rinaldi et al. [16] found a significant positive correlation between punch force and both right and left leg force in professional karate athletes by synchronizing the acquisition of kinematic, kinetic, and surface electromyographic (EMG) data.

Several studies have addressed the impact of karate training on muscular contractile properties. It has been well-established that following chronic exposure to specific training regimens, motor control programs undergo specific adaptations [17]. For instance, Zehr et al. [18] found that karate athletes produce higher ballistic action peak moment, peak rate of moment development and peak acceleration than their untrained counterparts during ballistic elbow extension actions. Moreover, a study by Marie-Ludivine et al. [19] showed a reduction in reaction times in the middle-aged group for both 6- and 12-month periods of karate training. Similarly, 5 months of adapted karate training improved this ability in elderly adult groups [20]. The findings by Jemili et al. [1] support the conclusion that specific karate training reduces the contraction time and modifies motor control patterns during punches and kicks. The results of this research were obtained by measuring EMG activity during the kizamawashi geri kick and the gyaku tsuki punch before and after 3 months of intense karate training [1]. Research by Probst et al. [21] indicated that karate athletes have sport-specific adaptations in certain caudal strength and flexibility measurements. Likewise, Zago et al. [22] concluded that elite masters possess higher dynamic balance ability than non-elite karate practitioners.

Sport-specific requirements of point karate are conditioned with the fine tuning of the agonist and antagonist muscles [1,5,17,23]. In competition, the best means for scoring points involve striking first, performing selected technical combinations such as punching or kicking. The assailant takes the initiative to make the distance shorter and to strike rapidly. Horizontal stepping is a specific karate displacement that relies on contraction velocity of the agonist muscles (knee extensors). In other words, maximal velocity and explosive strength are the major muscle mechanical factors involved in karate performance [9]. The quadricep muscle group acts as an agonist not only in stepping towards the opponent while punching, but also in fast kicking techniques. For instance, a study by Pozo et al. [24] found that the duration of the mae geri (globally used Japanese terminology for the front kick (FK)) was significantly shorter for international than for national-standard athletes. On the other hand, besides the high movement velocity, karateka often need to exert a strong braking action with the antagonist muscle groups to avoid the impact between the punching hand or kicking leg and the opponent’s body, as requested by the international rules for most technical actions [25]. The activity of the antagonist muscle increases proportionally to the intensity of the movement, the so-called co-contraction. A greater co-contraction implies higher antagonist muscle opposition to the intended movement [26]. The hamstring muscles act as antagonists in most karate actions.

Despite many studies that have addressed the voluntary muscle contractions of karate practitioners, there is a gap in the research of involuntary (stimulated) muscle contraction characteristics of this population, which is essential for advancing our understanding of this topic. Specifically, measurement of involuntary mechanical and contractile muscle properties across a variety of conditions, without violating daily training routine and avoiding the influence of factors such as motivation, fatigue or injuries is of utmost importance. Thus, the aims of the study were: (i) to reveal if essential differences in involuntary neuromuscular characteristics between top-level karate athletes and the non-athletes exist and if so, (ii) to determine to which extent the muscle functions can be defined by this method. In order to answer these questions, the TMG characteristics of the two groups flexor and extensor knee muscles were compared. We hypothesized that top-level karate athletes would show neuromuscular differences in involuntary neuromechanical contractile properties of the thigh muscles as a consequence of years of specific training and competing.

## 2. Materials and Methods

### 2.1. Participants

This study included a sample of 13 top-level karate athletes from the Serbian national team (KAs) and 14 non-athlete male individuals (NAs) (Table 1). The inclusion criteria for the KA group were: (1) Kumite fighters who had competed at the finals of the National Championship and higher-level competitions (European and World Championships); (2) all athletes involved in the study have master ranks in karate (black belts with a minimum of 1st dan). Subjects were included in the NA group if they had never practiced karate but did take part in physical activities 2–3 times per week at a noncompetitive level. Most of the NAs indicated their right leg as the dominant one (12), while only 2 people were left-footed. In the KA group, the dominant leg was self-reported as the leg used in order to lead out in movement, while the non-dominant leg was defined as the leg which performs the stabilizing or supporting role [27]. Specifically, the dominant leg was defined as the pushing leg in the fighting stance and at the same time the one that was reported to be most often used in kick-scoring techniques. The non-dominant one was the front leg that stops the movement, supports the kicking leg and controls the distance between the karateka and his opponent. The KA group had 10 right-leg-dominant and 3 left-leg-dominant athletes. None of the participants reported any medical problem or recent injuries that could compromise the tested performance. All participants were fully informed of the potential risks associated with the study and signed written informed consent forms previously approved by the Research Ethics Committee of the Faculty of Sport and Physical education, University of Belgrade (No. 484-2), in line with the criteria of the Declaration of Helsinki for research involving human beings.

### 2.2. Procedures

The participants were tested in the morning and had not practiced physical activity in the 24 h preceding the testing. All tests were performed by a team of researchers which included three experienced persons. The experiment was carried out within a single testing session. It included anthropometric measurements followed by the TMG assessment. Body height (BH) and body mass (BM) were measured to the nearest 0.1 cm and 100 g, respectively. Thereafter, the body mass index was calculated (BMI = BM/BH^2^).

The involuntary contractile characteristics of the lower-limb muscles—the RF, VM, VL, BF and ST—were recorded by measuring the response of these muscles to an induced electric stimulus (provoked by two self-adhesive electrodes) using TMG equipment (TMG-100 System electrostimulator, TMG-BMC d.o.o., Ljubljana, Slovenia) on both the right and left lower extremities. One muscle was tested on the right and the left leg, after which the focus was transferred to another muscle. The RF, VM and VL were assessed with the participants in the supine position, while the BF and ST were evaluated with the participants in the prone position. The knee joint was fixed at an angle of 120° [28]. The measuring location was carefully determined as the point of maximal muscle belly displacement during the voluntary contraction [29]. Between the electrodes, a sensor was positioned (GK40, Panoptik, Ljubljana, Slovenia) that detected changes in the muscle belly caused by electrical stimulations, based on which data are obtained on the involuntary muscle contractile properties [28]. All measurements were performed by the same expert technician, applying six electrical stimuli for 1 ms (10, 25, 50, 75, 100, and 110 mA). Ten seconds of recovery between measurements were allowed to minimize the effects of potentiation and fatigue. From each participant, only the curve that obtained the greatest Dm (maximum radial displacement of the muscle belly) was considered for analysis in each assessed muscle [11].

Group comparison was applied in order to assess the differences in contractile properties relative to the muscle role in the knee joint of KAs’ and NAs’ dominant and non-dominant legs (extensors and flexors, i.e., agonists and antagonists). The variables assessed in this study were the maximal radial displacement of the muscle belly (Dm), contraction time (Tc), delay time (Td), total contraction time (Tct) and rate of muscle tension development (RMTD). The Tc is obtained by determining the time lapse from 10% to 90% of Dm. The Td represents the time spent to reach 10% of the total movement after stimulation. The Tct was calculated as the sum of Tc and Td. The TMG-derived RMTD was not obtained through direct measuring, but instead was calculated as the relation between Dm and Tc and expressed in mm/ms [30]. All tests were conducted at the Faculty of Sports and Physical Education, University of Belgrade.

### 2.3. Statistics

Descriptive analysis was applied to describe the quantitative measures for the studied variables (Mean, SD), while multivariate analysis of variance (MANOVA) was performed to determine the differences between the groups (dominant and non-dominant leg flexors/extensors muscles in the knee joint). For determining the differences in individual TMG parameters, a T-test was applied along with the Bonferroni correction. Cohen’s d effect size was calculated to assess the practical significance of the findings. ES was interpreted as follows: *d* < 0.2 (trivial), *d* = 0.20–0.49 (small), *d* = 0.50–0.79 (medium) and *d* ≥ 0.80 (large) [31]. The level of statistical significance was set at *p* < 0.05 [32], while all the statistical procedures were performed on the SPSS-v20 program (IBM, Chicago, IL, USA).

## 3. Results

On a general level, there were significant differences between the dominant leg extensor muscles, dominant leg flexor muscles and non-dominant leg flexors between KAs and NAs, while there were no differences in non-dominant extensors between the observed groups (Table 2).

On the level of individual muscles, there were significant differences between the KA and NA in the RF, VM and VL knee extensor muscles (Table 3) and in the BF and ST knee flexor muscles (Table 4).

**Figure 1:**
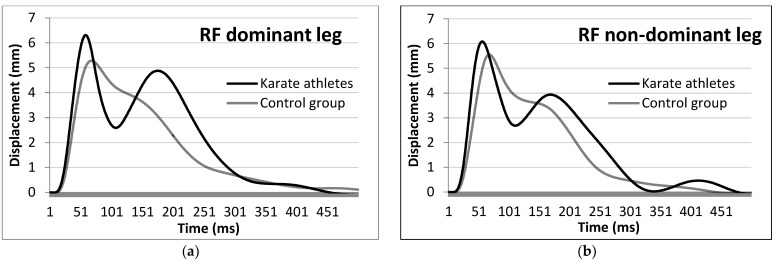
Average rectus femoris muscle response of the KAs’ and NAs’ dominant (**a**) and non-dominant (**b**) legs to an electric stimulus by means of TMG.

KAs showed lower Td, Tc and Tct values in the non-dominant leg (−2.48 ms, Cohen’s *d* = 0.909 for Td; −5.99 ms, Cohen’s *d* = 1.092 for Tc and −8.47 ms, Cohen’s *d* = 1.145 for Tct) and higher RMTD values of the RF muscle in the dominant leg (+0.07 mm/ms, Cohen’s *d* = 0.823) than the NAs (Figure 1).

**Figure 2:**
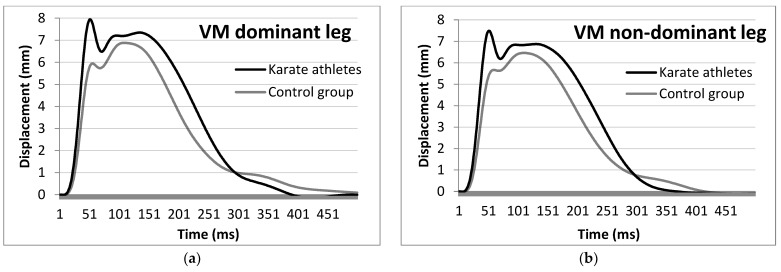
Average vastus medialis muscle response of the KA and NA dominant (**a**) and non-dominant (**b**) leg to an electric stimulus by means of TMG.

KAs also demonstrated significantly lower Td, Tc and Tct and higher RMTD values in the VM muscle of the non-dominant leg (−2.01 ms, Cohen’s *d* = 1.224 for Td; −5.28 ms, Cohen’s *d* = 1.096 for Tc, −7.29 ms, Cohen’s *d* = 1.236 for Tct and +0.11 mm/ms, Cohen’s *d* = 1.091 for RMTD), lower Tct, and higher Dm and RMTD values in the dominant leg (−6.38 ms, Cohen’s *d* = 0.924 for Tct; +1.56 ms, Cohen’s *d* = 0.831 for Dm and +0.1 mm/ms, Cohen’s *d* = 1.056 for RMTD) than the NAs (Figure 2).

**Figure 3:**
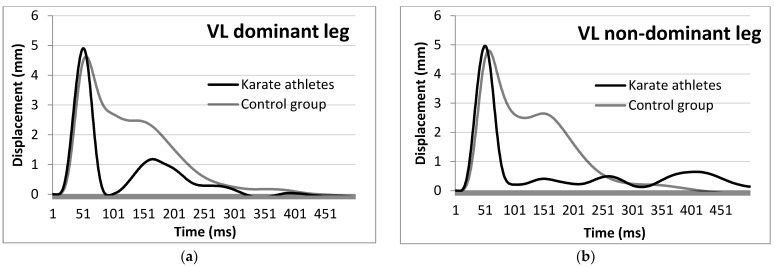
Average vastus lateralis muscle response of the KA and NA dominant (**a**) and non-dominant (**b**) legs to an electric stimulus by means of TMG.

KAs showed lower Td, Tc and Tct values in the VL muscle in both non-dominant and dominant leg when compared to the NAs (−2.44 ms, Cohen’s *d* = 1.211 for Td; −2.58 ms, Cohen’s *d* = 0.908 for Tc and −5.02 ms, Cohen’s *d* = 1.181 for Tct of the non-dominant leg, while −2.77 ms, Cohen’s *d* = 1.426 for Td; −2.65 ms, Cohen’s *d* = 0.889 for Tc and −5.41 ms, Cohen’s *d* = 1.310 for Tct of the dominant leg) (Figure 3).

**Figure 4:**
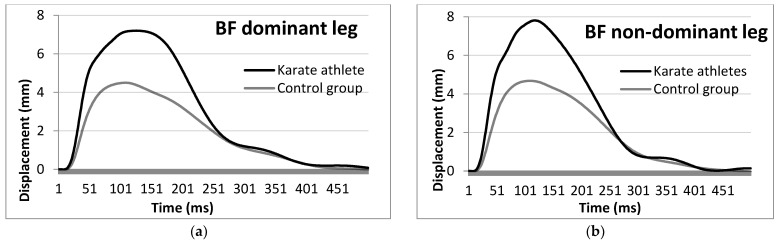
Average biceps femoris muscle response of the KA and NA dominant (**a**) and non-dominant (**b**) legs to an electric stimulus by means of TMG.

Regarding the BF, the KA group showed significantly higher RMTD in the non-dominant leg (+0.07 mm/ms, Cohen’s *d* = 0.984), as well as Dm and RMTD in the dominant leg (+2.45 mm, Cohen’s *d* = 0.906 for Dm and +0.06 mm/ms, Cohen’s *d* = 1.320 for RMTD) when compared to the NA group (Figure 4).

**Figure 5:**
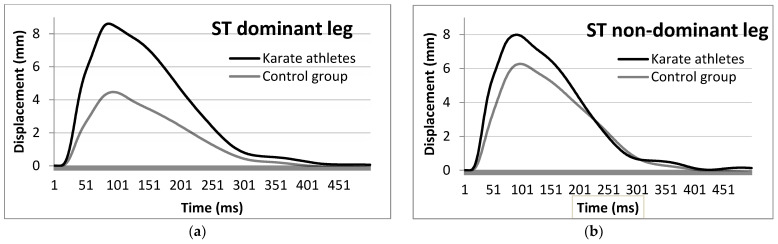
Average semitendinosus muscle response of the KA and NA dominant (**a**) and non-dominant (**b**) legs to an electric stimulus by means of TMG.

In KAs, the ST muscle of the non-dominant leg showed significantly lower values in Tct, but higher in RMTD (−11.04 ms, Cohen’s *d* = 0.935 for Tct and +0.07 mm/ms, Cohen’s *d* = 1.055 for RMTD), while the dominant leg showed significantly higher values for Dm and RMTD (+3.98 mm, Cohen’s *d* = 1.656 for Dm and +0.1 mm/ms, Cohen’s *d* = 1.859 for RMTD) compared to the control (Figure 5).

## 4. Discussion

Within the present article, we investigated whether there are differences between the neuromuscular profiles of top-level karate kumite athletes and non-athlete male individuals. The evaluated involuntary contractile properties of selected lower-limb muscles (RF, VM, VL, BF and ST) were Td, Tc, Tct, Dm and RMTD. Significant differences were found on a general level between the dominant leg extensors, dominant leg flexors and non-dominant leg flexors. With respect to individual variables, 25 differences were identified. A shorter Td, Tc and Tct in the knee extensor muscles (agonists), and higher values of Dm and RMTD were found in the knee flexor muscles (antagonists) of KA.

Significant differences on a general level between dominant knee joint extensors were mainly influenced by the differences in Tct of VM and VL. This suggests that agonist muscles of the dominant KA’s leg generally have different levels of twitch force generation speed, muscle responsiveness, and muscle fiber composition [28,33]. On the other hand, significant differences between dominant knee flexors were influenced by Dm and RMTD of the BF and ST, while non-dominant knee flexors were mainly influenced by differences in the RMTD variable of BF and ST muscles. Higher RMTD values suggest strength characteristics in the breaking action of the antagonist hamstring muscles, due to the specific learning strategies adopted in order to better control the kicking impact imposed by the karate competition rules [24,25].

Regarding the results of the TMG, KAs showed a great ability to rapidly generate force during involuntary contractions (Td and Tc lower than 30 ms) [34,35]. When compared to the NAs, KAs showed significantly lower Td values in the RF (non-dominant leg), VL (dominant and non-dominant leg) and VM (non-dominant leg) muscles. The Td results are quite similar to those found in the RF and BF muscles of elite and subelite futsal players [35], VM and BF of professional soccer players [36] and VM of elite amateur road cyclists [37]. The Tc parameter of the non-dominant RF, both dominant and non-dominant VL and non-dominant VM muscles showed significant differences when compared with the NAs. Likewise, a study by Garcia et al. [38] showed similar values of Tc of the RF and VL muscles in elite wrestlers. Tct was found to be significantly lower in KAs (RF of the non-dominant leg, dominant and non-dominant VL, dominant and non-dominant VM and ST of the non-dominant leg) than in the NAs. The VM of the KAs’ dominant legs also had significantly lower values in Tct, despite the fact that neither the Td nor Tc were significantly different. Td, Tc and Tct differences suggest that KAs are more explosive than the NAs and they have a greater number of type 1 fibers [39,40]. These results are in accordance with the fact that athletes who regularly take part in planned physical activities as a consequence benefit from adaptation to the training [41], which is manifested in a greater speed of contraction than in the NA group. On the other hand, significant differences were mostly found in the Dm and RMTD of the KA antagonist muscle group (knee flexors). The Dm values of the BF, ST and VM (dominant leg) muscles were higher than in the NAs, which is in contrast to studies showing that lower Dm values are associated with greater exposure to strength, power or plyometric training [42,43]. However, karate training and competition demands are proved to increase lower limb muscle flexibility [21,44], so this impact contributes to higher amplitudes of muscle displacement in this specific TMG variable. Similar values of Dm are found in professional soccer players’ BF [36], elite amateur road cyclists’ BF muscles [37] and elite futsal players’ RF [35]. The ability of muscles to achieve the most intense possible contraction was also found to be significantly higher in top-level karateka than in the NAs. KAs have a greater RMTD than the NAs in the case of the BF (dominant and non-dominant leg), ST (dominant and non-dominant leg), RF (dominant leg) and VM (dominant and non-dominant leg) muscles.

Top-level karateka’s agonist muscles (VL, VM and RF) Td, Tc and Tct are found to be different than in the control group. The habituation to ballistic exercises in these athletes has most probably induced specific neuromuscular changes in muscle responsiveness [45]. The examined VL and VM muscles are extensors in the knee joint [46], while the RF is an extensor in the knee and also a flexor in the hip joint [47]. Specifically, the RF muscle performs both of these actions during a kick by increasing the speed of the knee and reducing the time of lifting the foot up from the ground [13,48]. Since both hip flexion (jap. Hiki ashi) and knee extension movements are used in karate when executing kicks, the RF developed different contraction qualities than other muscles from the knee extensor group. These differences are reflected in a specific TMG record based on the automaticity of the RF double activation during leg karate techniques. As shown in Figure 1a,b, the complexity of the kicks’ performance generated two distinct contractile activities on the muscles in both dominant and non-dominant legs and in all karate practitioners, unlike the control group. KAs’ RF muscles activate in a double peak pattern, illustrating the relaxation phase after the foot leaves the surface (silence between the pulses on the KA signal group). This pattern is highlighting the importance of the ability to contract, relax and contract again quickly. The first peak of RF muscle activity is associated with initiation of motion as the hand [49] or foot [50] gained speed, while a relaxation phase is observed following the second peak associated with the body stiffening at impact (in case of strikes to the body) or energy exertion—jap. Zanshine (in case of strikes to the head). Studies that examined voluntary strikes with the EMG method also showed double muscle activation in elite mixed martial arts fighters [51] and karate athletes [52].

In the present study, the RMTD characteristic of antagonist muscles is found to be significantly higher in both examined hamstring muscles of KA (BF and ST) when compared to the NA. The RMTD difference of the antagonist muscles is based on previous experience acquired through years of practice and represents a centrally programmed anticipatory mechanism that controls the impact force and stabilizes the knee joint prior to impact. Kumite competitors demonstrate a great ability to activate or deactivate their antagonists in order to stabilize and protect the joint during kicks and punches [5,53]. Karate FK and roundhouse kick (RK) actions were investigated from an electrophysiological standpoint because of their strength [54] and predominant use in combat competitions [55], respectively. For instance, higher antagonist activation has been observed in elite karateka compared with amateur counterparts when asked to perform the FK, adopted to obtain a better control of the kicking leg [5]. Higher eccentric activation at the end of the attack phase is considered a pre-activation anticipating the knee flexion and the hip extension of the return phase [24]. In addition, higher BF antagonist activation was observed during the knee extension phase while performing the RK in senior than in junior karate competitors [17]. In this case, antagonist activation was significantly related to age, which, taken together with the previous study, points towards our hypothesis about the impact of specific karate training and its duration on the adaptation of muscular contractile properties.

Our findings offer a novel perspective on the muscle contractile properties of top-level karate competitors. Specifically, our results established values of the main muscles involved in karateka’s movement and kicking actions that could serve as a reference to training experts for monitoring karate athletes in various periods of macrocycle. This method of monitoring does not cause fatigue as with stress testing, so it does not alter the periodization of training. In addition, we presented the TMG signals of individual muscles, which provided insights of contraction–relaxation–contraction pulses that were highlighted only in a few combat sports studies assessed with the EMG method. The findings provided in this study should be considered in the framework of the following issues/limitations: (1) Only subjects of one gender were analyzed, and (2) this study did not analyze karate athletes at different competitive levels, i.e., training experience.

## 5. Conclusions

Based on the obtained results, we can conclude that on a group level, male karate competitors’ dominant-leg extensors and flexors and also non-dominant-leg knee flexors significantly differ from those of non-athletes. Additionally, differences were determined for 25 individual variables of the involuntary functional neuromechanical contractile properties of the thigh muscles. The results also indicated that these differences are very specific since they refer to specific characteristics and certain muscle groups. The TMG parameters in which the karate athletes and non-athletes differ the most are lower Tct in the case of the extensor muscles and higher RMTD in the flexor muscles of the karate athletes’ knee joint. The Tct values observed are suggestive of an improved ability of karateka to react fast with the quadricep muscle group of both legs, while the RMTD values suggest the enhanced contraction–relaxation cycle of the hamstring muscles, also in both legs. Findings of the RF muscle signals’ specificity confirms the production of a “double pulse”, more precisely the “contraction-relaxation-contraction” cycle, which opens new areas of training methods for improving striking efficiency. The TMG method is found to be sensitive enough to detect the neuromuscular adaptations induced by specific karate training. Future research may extend this work by an insight into the stimulated muscular contractile characteristics of female karate athletes and the differences between dominant and non-dominant legs, as well as the aforementioned characteristics during different macrocycle phases.

## Figures and Tables

**Table 1:** Descriptive characteristics of the study participants.

Variables	Karate Athletes					Non-Athletes				
	Mean	SD	cV%	Min	Max	Mean	SD	cV%	Min	Max
Age	24.08	4.50	18.69	18.00	32.00	26.07	3.75	14.39	19.00	30.00
BH (cm)	180.21	9.11	5.05	161.40	193.10	180.70	7.23	4.00	170.50	196.00
BM (kg)	79.51	8.07	10.14	63.00	92.20	82.77	13.30	16.07	60.80	106.30
BMI (kg/m^2^)	24.45	1.43	5.84	22.10	28.00	25.28	3.44	13.61	19.30	31.80

Note: BH—body height; BM—body mass; BMI—body mass index; SD—standard deviation; cV%—percent coefficient of variation; Min—minimum; Max—maximum.

**Table 2:** General differences between knee joint muscle groups—MANOVA.

Wilks’ Lambda	Value	F	df	Error df	Sig	Partial Eta Square	Observed Power
Dominant Extensors	0.308	2.625	12	14	0.044	0.692	0.789
Dominant Flexors	0.402	3.347	8	18	0.016	0.598	0.878
Non-dominant Extensors	0.307	2.255	13	13	0.078	0.693	0.703
Non-dominant Flexors	0.361	3.978	8	18	0.007	0.639	0.933

**Table 3:** Descriptive indicators (Mean ± SD) of the TMG variables and Student’s *t*-test results of the knee joint extensor muscles among male KAs and NAs.

		Dominant Leg		Non-Dominant Leg	
		Karate Athletes	Non-Athletes	Karate Athletes	Non-Athletes
		Mean ± SD	Mean ± SD	Mean ± SD	Mean ± SD
RF	Tc	28.84 ± 5.49	32.15 ± 6.31	25.37 ± 5.26	31.36 ± 5.71 *
	Td	22.34 ± 1.62	22.30 ± 2.92	22.18 ± 2.32	24.66 ± 3.13 *
	Tct	51.18 ± 6.42	54.45 ± 7.94	47.55 ± 7.04	56.02 ± 7.76 *
	Dm	6.60 ± 2.01	5.55 ± 2.16	6.42 ± 2.38	5.78 ± 2.28
	RMTD	0.24 ± 0.09	0.17 ± 0.07 *	0.26 ± 0.10	0.19 ± 0.08
VM	Tc	23.74 ± 2.87	28.19 ± 9.34	23.74 ± 2.64	29.02 ± 7.01 *
	Td	21.23 ± 1.14	23.16 ± 3.88	20.96 ± 1.62	22.97 ± 1.66 *
	Tct	44.97 ± 3.59	51.35 ± 10.23 *	44.70 ± 3.58	51.99 ± 8.22 *
	Dm	8.19 ± 2.13	6.63 ± 1.63 *	7.72 ± 2.60	6.02 ± 1.97 *
	RMTD	0.35 ± 0.09	0.25 ± 0.09 *	0.33 ± 0.11	0.22 ± 0.09 *
VL	Tc	23.09 ± 2.12	25.74 ± 3.83 *	22.91 ± 2.52	25.49 ± 3.16 *
	Td	21.46 ± 0.76	24.23 ± 3.12 *	20.66 ± 1.85	23.10 ± 2.18 *
	Tct	44.56 ± 2.61	49.97 ± 5.66 *	43.57 ± 4.04	48.59 ± 4.46 *
	Dm	5.02 ± 1.50	5.00 ± 2.44	5.15 ± 1.67	5.14 ± 1.47
	RMTD	0.22 ± 0.06	0.20 ± 0.10	0.22 ± 0.06	0.21 ± 0.08

Note: * Significance at *p* < 0.05 level; RF—rectus femoris; VM—vastus medialis; VL—vastus lateralis; Tc—contraction time; Td—delay time; Tct—total contraction time; Dm—maximal radial displacement of the muscle belly; RMTD—rate of muscle tension development. Parameters are expressed in milliseconds (Tc, Td and Tct), millimeters (Dm) and millimeters/milliseconds (RMTD).

**Table 4:** Descriptive indicators (Mean ± SD) of the TMG variables and Student’s *t*-test results of the knee joint flexor muscles among male KAs and NAs.

		Dominant Leg		Non-Dominant Leg	
		Karate Athletes	Non-Athletes	Karate Athletes	Non-Athletes
		Mean ± SD	Mean ± SD	Mean ± SD	Mean ± SD
BF	Tc	32.22 ± 11.31	35.20 ± 9.38	41.57 ± 14.97	36.25 ± 9.65
	Td	22.93 ± 1.95	26.49 ± 10.39	23.24 ± 2.13	22.98 ± 5.05
	Tct	55.15 ± 13.06	61.69 ± 15.28	64.81 ± 16.59	59.23 ± 14.11
	Dm	6.53 ± 1.94	4.82 ± 1.83 *	7.43 ± 2.52	4.98 ± 3.09
	RMTD	0.21 ± 0.06	0.14 ± 0.05 *	0.19 ± 0.07	0.13 ± 0.06 *
ST	Tc	41.39 ± 12.11	35.70 ± 13.34	38.28 ± 13.70	47.30 ± 6.91
	Td	24.01 ± 1.83	25.21 ± 8.06	23.10 ± 2.59	25.11 ± 2.51
	Tct	65.40 ± 13.60	60.90 ± 14.94	61.38 ± 16.13	72.42 ± 7.49 *
	Dm	8.57 ± 1.95	4.59 ± 2.86 *	7.99 ± 2.85	6.58 ± 3.23
	RMTD	0.22 ± 0.05	0.12 ± 0.05 *	0.21 ± 0.05	0.14 ± 0.08 *

Note: * Significance at *p* < 0.05 level; BF—biceps femoris; ST—semitendinosus.

## Data Availability

The data are available on request to the corresponding author.
